# Inferior Rectus Myositis After Orbital Fracture Repair With the Medpor® Implant in a 38-Year-Old Male Patient

**DOI:** 10.7759/cureus.110763

**Published:** 2026-06-13

**Authors:** Thomas Ting Hei Tsang, Fatema Aljufairi, Jake U Sebastian, Kenneth Lai, Kelvin Chong

**Affiliations:** 1 Department of Ophthalmology and Visual Sciences, The Chinese University of Hong Kong, Hong Kong, HKG; 2 Department of Ophthalmology, Salmaniya Medical Complex, Government Hospitals, Manama, BHR; 3 Department of Ophthalmology, Vicente Sotto Memorial Medical Centre, Cebu, PHL

**Keywords:** floor fracture, inferior rectus, medpor implant, myositis, orbital fractures

## Abstract

We report a rare case of inferior rectus myositis related to a Medpor® orbital implant (Stryker Corporation, Kalamazoo, MI, USA) following orbital fracture repair. A 38-year-old gentleman, who underwent uneventful orbital floor fracture repair using a Medpor® implant, presented a week later with a dull ache and acute onset of diplopia on up- and downgaze. The symptoms were gradually relieved after a secondary transorbital, transantral biplanar surgical approach for removal of an orbital Medpor® implant and repair of the orbital fracture. Despite the high success rates seen with Medpor® orbital implants, the body may recognize the implant as foreign and can mount an immune response against it.

## Introduction

Orbital fractures are common maxillofacial injuries resulting from blunt facial trauma, with reported prevalence rates ranging from 3% to 32% of facial fractures [1]. Clinical consequences include extraocular muscle restriction, diplopia, hypoesthesia, enophthalmos, and cosmetic deformity [2]. Surgical repair is often indicated in cases of persistent diplopia, significant enophthalmos, large orbital floor defects, or radiologic evidence of tissue entrapment [3].

Medpor® (Stryker Corporation, Kalamazoo, MI, USA) is a porous high-density polyethylene implant that has been widely used in orbital wall and rim reconstruction for more than three decades [4]. Its popularity stems from its biocompatibility, ease of contouring, structural stability, and ability to permit fibrovascular ingrowth through its interconnected porous architecture. Although Medpor® implants generally demonstrate excellent long-term outcomes, reported complications include infection, migration, exposure, extrusion, fibrosis, orbital adherence syndrome, and persistent diplopia [5].

Orbital myositis is an inflammatory condition affecting one or more extraocular muscles and typically presents with diplopia, ocular discomfort, motility restriction, and enlargement of the involved muscle on imaging. While most cases are idiopathic or associated with systemic inflammatory, infectious, or autoimmune conditions, implant-associated extraocular muscle inflammation is exceedingly rare [6,7]. The proposed mechanisms include chronic foreign-body inflammatory reaction, fibrosis, soft-tissue adherence, and direct entrapment of orbital tissues adjacent to the implant.

We report a rare case of isolated inferior rectus myositis following orbital floor fracture repair with a Medpor® implant. The patient developed persistent diplopia and motility restriction associated with radiologic evidence of inferior rectus involvement that was refractory to medical therapy. Management required revision surgery using a biplanar transorbital-transantral approach for implant removal and reconstruction. This case highlights a rare but clinically important complication of porous polyethylene implants and demonstrates a surgical strategy for managing implant-associated inferior rectus inflammation. Patient confidentiality was respected in accordance with the tenets of the Declaration of Helsinki.

This article was previously presented as a poster at the 35^th ^Annual Scientific Meeting of the Hong Kong Ophthalmological Symposium.

## Case presentation

A 38-year-old Chinese male who was a non-smoker and unknown to have any systemic illness underwent a repair of a left orbital floor fracture using a Medpor® orbital implant elsewhere, one day after sustaining facial trauma. Intractable diplopia was reported one week postoperatively. A course of oral amoxicillin/clavulanate 1 g twice daily for a week was given to the patient. Persistence of diplopia prompted evaluation with orbital computed tomography, which showed an isolated nodular enlargement of the inferior rectus muscle (Figure 1).

**Figure 1:**
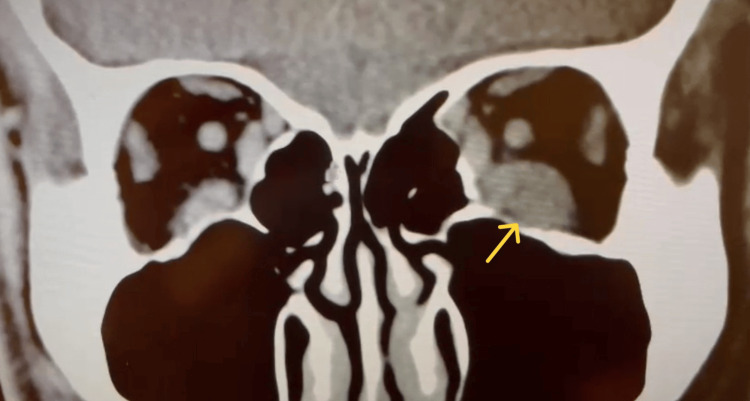
Computed tomography (coronal section) showing enlarged left inferior rectus myositis (arrow).

Oral prednisolone (1 mg/kg/day) and prophylactic antibiotics were prescribed to manage the myositis. Non-resolution of the patient’s symptoms resulted in a referral to our institution.

The patient presented to our center three weeks postoperatively with persistent diplopia and a marked dull ache in the left orbit. On examination, visual acuity measured using a logMAR chart was 0.18 in the right eye and 0.00 in the left eye. Left hypotropia and intractable diplopia in primary and upgaze positions were noted, with extraocular motility restriction of −3 in upgaze and −2 in downgaze. Hertel exophthalmometry demonstrated 18 mm and 20 mm for the right and left eyes, respectively. Serologic testing revealed a thyroid-stimulating immunoglobulin level of <89 and a positive antinuclear antibody test.

Although autoimmune orbital inflammation and thyroid eye disease were considered in the differential diagnosis, the unilateral focal muscle involvement adjacent to the implant, absence of characteristic radiologic features of thyroid eye disease, and temporal association with recent orbital fracture repair favored an implant-associated inflammatory and mechanical process. Multiparametric dynamic orbital MRI demonstrated focal enlargement and T2 hyperintensity of the inferior rectus muscle, together with adjacent soft-tissue entrapment secondary to incomplete defect coverage. Dynamic T2 cine sequences confirmed restricted elevation. Collectively, these findings were suggestive of a combined inflammatory and mechanical process involving the inferior rectus muscle rather than isolated myositis alone (Figures 2, 3).

**Figure 2:**
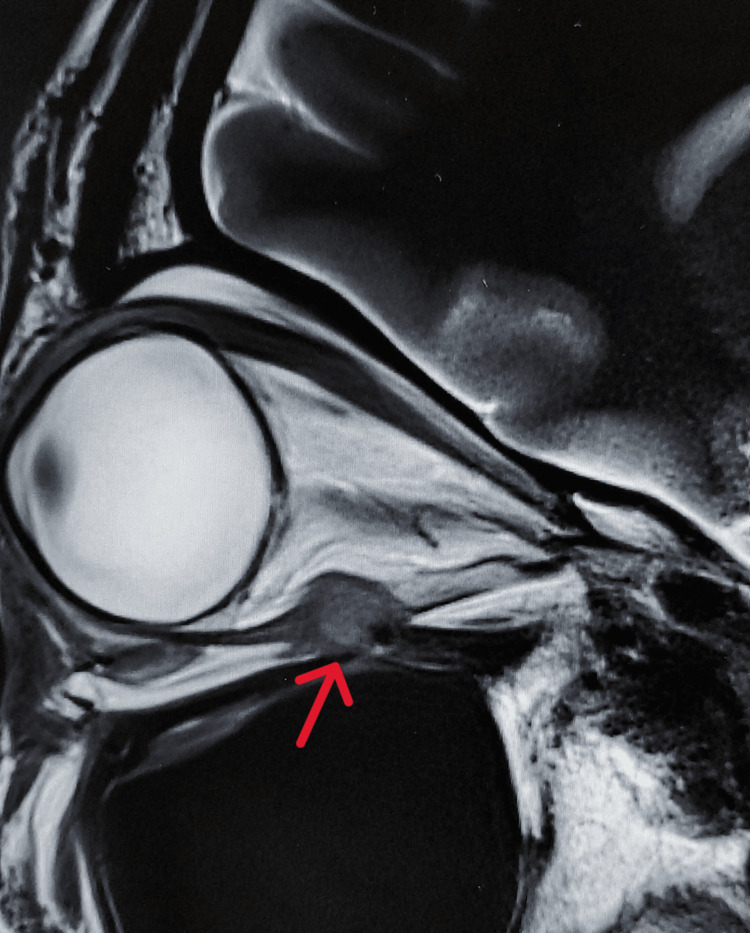
MRI (sagittal section T2-cine sequence) showing nodular enlargement of the inferior rectus muscle belly (red arrow).

**Figure 3:**
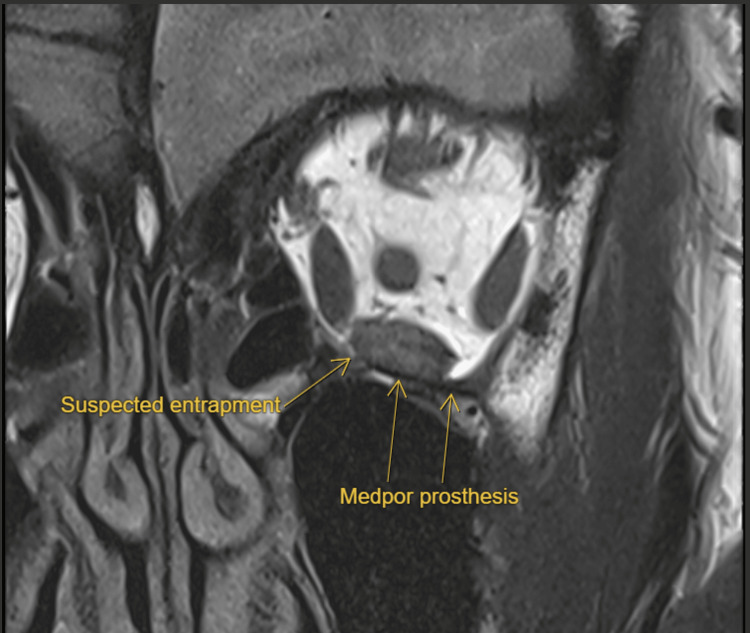
MRI (coronal section) showing the Medpor® implant and the medial area of suspected left inferior rectus muscle entrapment.

A joint decision for a revision surgery was made for orbital floor fracture surgery using a biplanar transorbital and transantral approach [4] with release of entrapped soft tissues, removal of orbital implant, and replacement with an autologous dermis fat graft.

The surgical procedure was performed under general anesthesia with the patient in the supine position. The left forced duction test was positive on upward traction and was negative for the right. The anterior sinus wall was exposed through a 1.5 cm intraoral upper buccal vestibular incision (Figure 4A), and a maxillary window (Figure 4B) was created using a 6 mm Medtronic high-speed drill (Medtronic plc, Dublin, Ireland). Transantral endoscopic evaluation of the orbital floor was done, noting healed sinus mucosa over the area of the fracture. Maxillary mucosal dissection through the transantral access employing a Kangaroo Pouch technique revealed the extent of the fracture, including the posterior edge, areas of adhesions, the Medpor® orbital implant with a medial area of non-coverage, and exposed inferior rectus muscle fibers (Figure 4C). The Kangaroo Pouch technique (Figure 4D) is a newly described pressure-driven submucosal dissection technique (by the corresponding author: KC) involving the use of transamine-soaked neurosurgical patties rolled into a submucosal pocket through a small incision. A straight periosteal dissector was used to apply gentle pressure on the neurosurgical patties, which reflected the mucosa en bloc off its attachment to bone, while the locally applied tranexamic acid in transamine provides much-needed hemostasis and offsets against trasamine-related systemic toxicity. A transconjunctival inferior forniceal incision with subperiosteal dissection (Figure 4E) was done to remove the Medpor® implant (Figure 4F) and to expose the orbital floor from above. A dermis fat graft was harvested from the anterior superior iliac crest (Figure 4G), was imbued with 5-fluorouracil, and positioned fat-side facing the orbit, to tamponade the floor defect (Figure 4H). The transconjunctival incision was allowed to heal by secondary intention.

**Figure 4:**
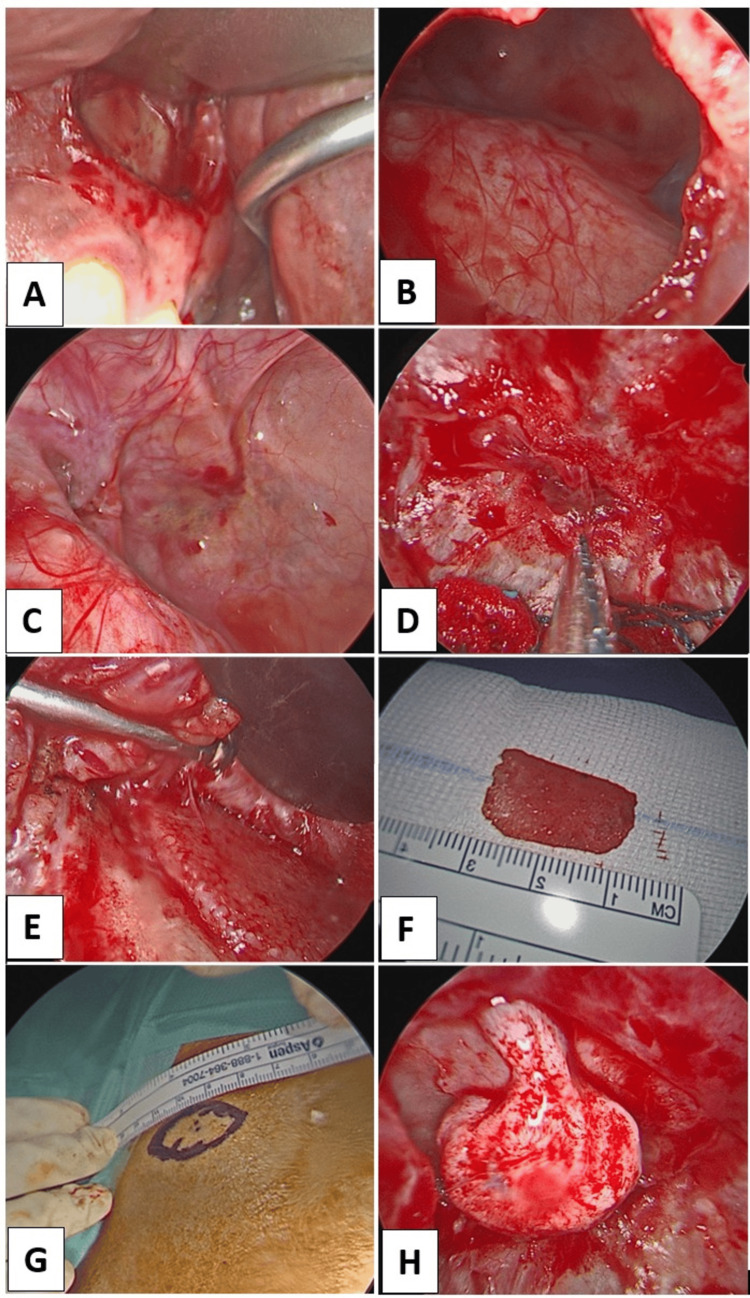
Intraoperative images A. 1.5-cm intraoral upper buccal vestibular incision; B.A maxillary window; C. Maxillary mucosal dissection through the transantral access; D. The Kangaroo Pouch technique; E. A transconjunctival inferior forniceal incision with subperiosteal dissection; F. Medpor® implant; G. A dermis fat graft was harvested from the anterior superior iliac crest. H. The orbital floor defect.

Complete coverage of the fracture site and absence of any entrapped soft tissues were ascertained by visualization through the transantral view. The buccal-gingival incision was closed with Vicryl sutures (Ethicon, Inc., Raritan, NJ, USA). A repeat forced-duction test at the end of the surgery was negative.

The patient was monitored serially postoperatively. A week after surgery, ocular alignment was straight in the primary position with upgaze diplopia in the left eye and excursions of (-1) upgaze and (-2) downgaze. Six months postoperatively, the visit revealed resolution of vertical diplopia, with only a (-1) upgaze limitation.

## Discussion

Orbital myositis is an inflammatory condition of the extraocular muscles that may be idiopathic or secondary to autoimmune disease, thyroid eye disease, infection, or trauma [8, 9]. In contrast, Medpor®-induced myositis is a rare complication. To the best of our knowledge, only one previous case report exists describing isolated inferior rectus myositis after an orbital fracture repair that was reported in 2017 by Kashkouli et al. on a seven-year-old female patient who had undergone an orbital floor blowout fracture repair with a Medpor® implant. The case was conservatively followed with an orbital CT, showing an isolated inferior rectus enlargement but not entrapment [10].

We report a similar case of Medpor®-related inferior rectus myositis that required revision surgery with removal of the Medpor® implant and replacement with an autologous dermis-fat graft. Alternative causes of orbital myositis were considered but were not supported by the clinical, laboratory, or radiologic findings. The persistence of gaze diplopia, radiologic evidence of soft-tissue entrapment (Figure 1C), and incomplete response of the entrapped, swollen inferior rectus muscle to systemic glucocorticoid and antibiotic therapy further supported a localized implant-related inflammatory process and prompted a more aggressive management approach.

Possible explanations for the constant upgaze and downgaze restriction after the first blowout fracture repair were residual entrapment of any orbital soft tissue, entrapment and necrosis of the inferior rectus muscle, or inferior rectus muscle fibrosis [11]. We can also deduce an inflammatory basis for the patient’s symptom of diplopia with an elevated anti-nuclear antibody upon further testing.

Orbit is an anatomically complex space, and modified surgical approaches have been described to improve exposure and visualization [12]. The biplanar transorbital and transantral approach allows for the precise determination of the orbital floor fracture size, fracture location, and evidence of entrapped soft tissues without artificially manipulating or modifying the orbital structures in situ. It also allows for direct evaluation of the posterior shelf of the orbital floor and assists with the reduction of complex orbital floor fractures.

In view of the myositis, as a result of entrapment and a possible negative reaction to the foreign orbital implant, an autologous suprailiac dermis-fat graft can be a suitable alternative to tamponade the defect and to augment the orbital volume.

Fat grafts are often the ideal choice when the allogenic implant is exposed or infected. Dermis fat grafts that induce less immunological reaction are easily obtained. A Korean study reported that Medpor® implantation with an orbital fat graft can effectively prevent orbital implant exposure [13]. The main concern of fat grafts is the unpredictability of atrophy. A Ghanian case series reported a success rate of 93.3% in using a dermis fat graft for volume replacement after enucleation, with preservation of orbital volume noted in 14 out of 15 patients over a five-year period [14].

Medpor® myositis involving the inferior rectus is a rare complication that can occur after an uneventful surgery. A history of orbital fracture repair with a Medpor® implant, postoperative diplopia, and motility restriction should raise the clinical suspicion of a surgical complication and should prompt investigation. Endoscopic-assisted techniques have increasingly been used in orbital and lacrimal surgery to improve visualization within anatomically constrained spaces [15]. Transantral endoscopic visualization during transconjunctival floor repair allows for a pressureless, precise identification of the posterior edge of the fracture and visualizes any entrapped tissue after implant insertion without retracting the orbit. Combining modalities of a Medpor® implant lined with a dermis-fat graft may reduce the development of adhesions and help with orbital volume augmentation. More research and development on proper surgical technique, improvement of implant material, and timely postoperative intervention can help reduce the risk of complications associated with the use of the Medpor® implant.

## Conclusions

Given the exceptionally low incidence of this complication, Medpor®-related myositis is unlikely to be a common cause of postoperative diplopia. Nevertheless, it should be considered in selected patients with persistent diplopia and motility restriction following orbital fracture repair, particularly when clinical and radiologic findings suggest implant-related extraocular muscle involvement. Transantral endoscopic visualization during transconjunctival floor repair allows for a pressure-less, precise location of the posterior edge of the fracture and visualizes any entrapped tissue after implant insertion without retracting the orbit. Combining modalities of a Medpor® implant lined with a dermis-fat graft may reduce the development of adhesions and help with orbital volume augmentation. More research and development on proper surgical technique, improvement of implant material, and timely postoperative intervention can help reduce the risk of complications associated with the use of the Medpor® implant.
